# From Genomic Fossils to Functional Elements: The Evolving Story of Pseudogenes

**DOI:** 10.1002/ggn2.202500040

**Published:** 2025-11-24

**Authors:** Mengyao Sun, Yanni Ma, Jia Yu

**Affiliations:** ^1^ State Key Laboratory of Common Mechanism Research for Major Diseases Key Laboratory of RNA and Hematopoietic Regulation Institute of Basic Medical Sciences Chinese Academy of Medical Sciences School of Basic Medicine Peking Union Medical College Beijing China; ^2^ Institute of Blood Transfusion Chengdu China

**Keywords:** evolution, gene duplication, non‐coding genes, pseudogenes, retrotransposition

## Abstract

Pseudogenes, as important products of genomic evolution, play unique regulatory roles in species adaptation. This review systematically summarizes the major types, functions, and regulatory mechanisms of metazoans pseudogenes, with a particular focus on their formation during primate evolution and the mechanisms underlying their retention in the human genome. Previous studies suggest that the loss of function in pseudogenes releases selective pressure, allowing them to evolve neutrally; furthermore, their latent functional or adaptive potential, such as reactivation, neofunctionalization, or evolutionary advantages conferred by gene silencing, further promotes their persistence. For instance, the integration of certain pseudogenes can introduce novel regulatory functions, while pseudogenization‐induced gene inactivation may also provide selective benefits. Recent technological advances, including long‐read sequencing, single‐cell omics, and CRISPR‐based functional interrogation, have greatly expanded our understanding of pseudogenes. We propose that pseudogene‐mediated regulation plays a critical role in evolutionary processes and highlight their dynamic roles in both physiological and pathological contexts. We further discuss current research progress, limitations, and future directions, offering new perspectives for understanding genomic evolution and biomedical significance of pseudogenes.

## Graphical abstract

This review begins with an introduction, summarizing the research background in pseudogene studies. It then contains three aspects: identification of pseudogenes, detailing their classification and features in metazoan genomes; functional pseudogenes, introducing their reactivation and regulation mechanisms; and pseudogenes’ contribution to species adaptation. Finally, the conclusion and perspective section highlights pseudogene functions, evolutionary significance, research challenges, and future directions.

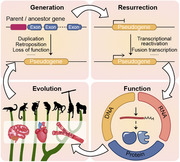

## 1 Introduction

The human genome has currently annotated over 50 000 genes, yet only 30% of them are protein‐coding genes [1]. A much larger proportion lacks protein‐coding capacity and is collectively referred to as non‐coding genes [2, 3]. Long non‐coding RNAs (lncRNAs), short non‐coding RNAs (including microRNAs, small nuclear RNAs, etc.), and pseudogenes constitute the major components of non‐coding genes. Among them, pseudogenes account for approximately one‐fourth of the entire human genome, second only to protein‐coding genes in number, making them a vast and highly important family of non‐coding genes [2, 3].

As early as 1977, Jacq et al. discovered the first pseudogene, a defective copy of 5S DNA, in Xenopus laevis [4], leading to the initial and fundamental definition of pseudogenes as truncated gene copies of functional genes [5]. However, for a long time afterward, despite the increasing identification of pseudogenes, their biological functions received little attention. Genomic regions annotated as pseudogenes were often excluded from functional screenings and genomic analyses [6, 7, 8, 9, 10]. It was only after the functional significance of non‐coding RNAs (such as lncRNAs and microRNAs) gained recognition that some pseudogenes were found to be reactivated, and capable of transcription and even protein translation. The transcripts or encoded products derived from pseudogenes may participate in regulating various biological processes, functioning under both physiological and pathological conditions [11, 12, 13, 14]. While most pseudogenes in the human genome exhibit high species specificity, with the vast majority being unique to higher primates, pseudogenes are generally considered to evolve under neutral selection, and thus far, only a few have been implicated in regulating species‐specific evolutionary processes [15, 16, 17].

In this review, we systematically outline the fundamental characteristics of metazoan pseudogenes, including their types and sequence features across species, and summarize the known mechanisms of pseudogene reactivation and function. Although pseudogenes have received limited attention in evolutionary studies, emerging evidence suggests that pseudogenes that mediated functional innovations may contribute to species evolution. Pseudogenes also possess potential cis‐regulatory elements and can even be functionally resurrected under specific selective pressures. Compared to previously published reviews on pseudogenes [18, 19, 20], our work incorporates recent research advances and emerging developments in the field, while also discussing and anticipating potential breakthroughs enabled by technological progress. More importantly, we provide a novel and rarely explored perspective, how pseudogenes may participate in species evolution, in what capacity, and through which mechanisms, offering a systematic synthesis of functional pseudogenes and their roles in evolutionary processes. In summary, we propose that functional pseudogenes may serve as a critical driving force in evolution, while the vast reservoir of currently silent pseudogenes with unknown functions may represent an essential genomic resource for evolutionary adaptation.

## 2 Identification and Classification of Pseudogenes

Pseudogenes are characterized primarily by their sequence similarity to parental genes and the presence of disabling mutations, such as frameshifts, premature stop codons, and so on [21]. To maximize the accuracy of pseudogene annotation, current methodologies combine computational pipelines with manual curation to distinguish pseudogenes from their functional counterparts [3, 22, 23]. Sequence alignments enable the inference of pseudogene origins, facilitating their annotation and classification.

### 2.1 Bioinformatic Approaches for Pseudogene Identification

Bioinformatic identification of pseudogenes is a multi‐step process that relies on integrated strategies, with the core challenge being the distinction between pseudogenes and their functional parental genes. The standard pipeline begins with initial homology‐based screening using tools such as BLAST or BLAT to align whole‐genome sequences or assembled transcripts against reference protein‐coding databases, enabling the detection of highly homologous yet potentially defective sequences as candidate pseudogenic regions. This is followed by the critical step of detecting inactivating mutations, such as premature stop codons, frameshift insertions and deletions (indels), splice site disruptions, and loss of the start codon, which serve as definitive evidence of loss of coding capacity [24]. Overall, annotated pseudogenes fall into two major categories based on the recognition features: processed pseudogenes, which specifically refer to those derived from parental gene transcripts through post‐transcriptional processing, typically representing retroposed pseudogenes, and unprocessed pseudogenes, which arise without such processing and generally encompass duplicated pseudogenes and unitary pseudogenes. Different types of pseudogenes are not only generated in different ways but also differ in sequence characteristics, conservation, and even functional preferences. Specialized tools, including PseudoFinder, RetroFinder, and PPFINDER, have been developed to systematically integrate these features for genome‐wide pseudogene annotation.

Evolutionary constraint analysis provides further support for pseudogene identification [21, 25]. Pseudogenes exhibit remarkable species specificity compared to protein‐coding genes, primarily manifested by their distinct evolutionary patterns and genomic characteristics. Beyond differences in sequence conservation, protein‐coding genes and pseudogenes are subject to fundamentally distinct selective pressures: protein‐coding genes experience strong purifying selection that efficiently eliminates non‐synonymous mutations disrupting protein structure or function, maintaining high sequence conservation across evolutionary timescales, while pseudogenes evolve predominantly through neutral processes, accumulating mutations at genomic background levels as “genomic fossils” that record evolutionary history without functional constraint. Thus, the calculation of nonsynonymous‐to‐synonymous substitution rate ratio (Ka/Ks) can serve as a proposed methodology for pseudogene identification and assessing the coding potential of putative pseudogenes, especially reliable for ancient, intact pseudogene copies that have accumulated sufficient substitutions while maintaining reading frame continuity with their progenitors.

Long‐read sequencing is also extensively applied in the discovery and annotation of pseudogenes. Through high‐quality long‐read assembly, this technology enables the identification of previously unannotated pseudogenes that are absent from the reference genomes of humans, great apes, and other species, making it a powerful tool for novel gene discovery [26, 27]. In sum, ongoing efforts such as the GENCODE project continuously refine gold‐standard pseudogene annotations mainly for human and mouse [3, 28]. Dedicated databases and resources, including Gerstein labs’ pseudogene database (http://www.pseudogene.org), psiDR (http://pseudogene.org/psidr/), and GenTree (http://gentree.ioz.ac.cn/) [3, 29, 30], provide comprehensive, curated information on pseudogenes across multiple species.

Consequently, the pseudogene repertoire is continually expanding and being refined, driven by advances in annotation software, the incorporation of evolutionary data, and the integration of long‐read sequencing technologies, providing a foundation for evolutionary and functional studies.

### 2.2 Classification of Pseudogenes in Metazoans

**FIGURE 1:**
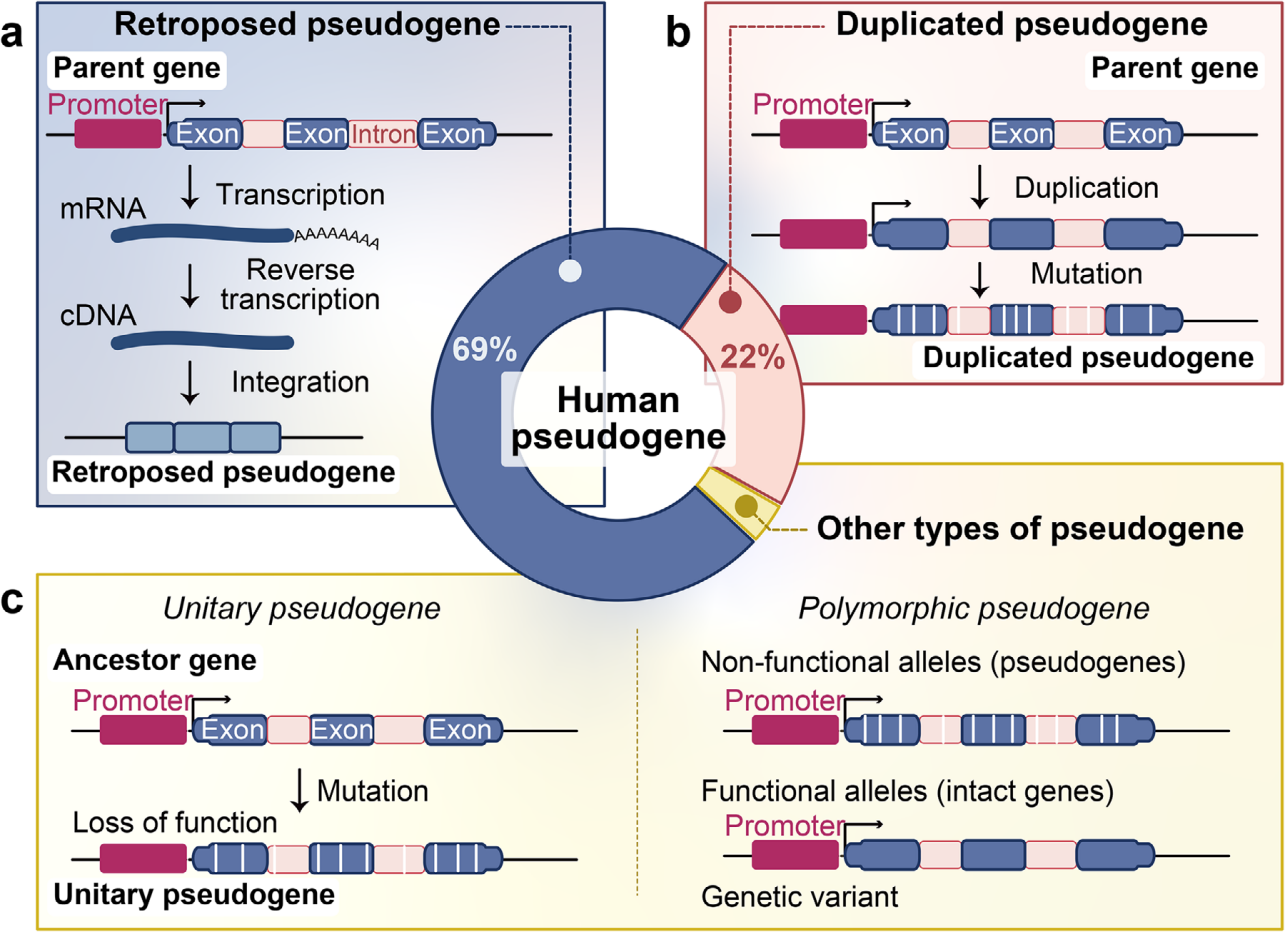
Main types and origins of human pseudogenes. (a) Retroposed pseudogenes (∼70% of total), generated via reverse transcription and genomic reintegration. (b) Duplicated pseudogenes (∼20%), produced by gene duplication followed by mutation accumulation. (c) Other pseudogene types: unitary pseudogenes (ancestral gene inactivation, left) and polymorphic pseudogenes (functional alleles coexisting with non‐functional variants in a population).

Building on relatively well‐established genome annotation tools, pseudogenes are primarily categorized into three major classes based on their mechanisms of formation: retroposed pseudogenes, duplicated pseudogenes, and unitary pseudogenes. Retroposed pseudogenes, also termed processed pseudogenes, are generated through retrotransposition‐mediated insertion of parental gene transcripts into new genomic loci (Figure 1a) [5, 31]. Human‐retroposed pseudogenes predominantly originated during the ancestral primate retrotransposition burst and currently represent the most abundant pseudogene class in the human genome, accounting for approximately 70% of all pseudogenes [32, 33]. Their formation is mechanistically linked to the transposition activity of LINE1 (L1) elements, which encode endogenous reverse transcriptases capable of co‐opting the transcripts originating from distinct genes for reintegration [34]. Consequently, most retroposed pseudogenes are characterized by the absence of introns, a hallmark feature distinguishing them from their parental genes [35, 36]. Notably, approximately 8.9% of protein‐coding genes are also devoid of introns and may have been generated by retrotransposons, suggesting retroposition as a significant pathway for novel gene origination [37]. Duplicated pseudogenes arise from direct genomic duplication events, retaining both intronic sequences and proximal regulatory elements from their parental genes (Figure 1b) [3]. Typically localized near their functional counterparts, duplicated pseudogenes exhibit high sequence similarity with their progenitors but are rendered nonfunctional through degenerative mutations. Derived from in situ inactivation of ancestral genes, unitary pseudogenes accumulate loss‐of‐function mutations while remaining genomically retained (Figure 1c) [38]. Although lacking functional parental genes in humans, orthologous functional genes often persist in other species. Additionally, there exist relatively rare pseudogene classes, such as polymorphic pseudogenes (Figure 1c), wherein a single genetic locus within a biological population concurrently harbors both functional alleles (intact genes) and non‐functional alleles (pseudogenes) [3, 39]. This genetic variant may represent an intermediate evolutionary state between gene functionality and complete pseudogenization. Furthermore, two rare pseudogene types exhibit unique origins and functions. circRNA‐derived pseudogenes originate from reverse‐transcribed circular RNAs and display non‐collinear exon arrangements relative to parental genes, potentially influencing genomic structural remodeling [40, 41]. Chimeric pseudogenes, formed by fusion of sequences from multiple genomic sources, highlight the complexity of genomic rearrangements and serve as valuable models for studying evolutionary innovation and structural diversity [42].

In sum, regardless of whether through retrotransposition, duplication, or in situ mutation and so on, pseudogenes typically lose protein‐coding capacity during their generation through various molecular defects, including aberrant splicing, frameshift mutations, or premature termination codon introduction, ultimately becoming defective genes on the genome.

### 2.3 Characteristics of Major Pseudogene Types

**FIGURE 2:**
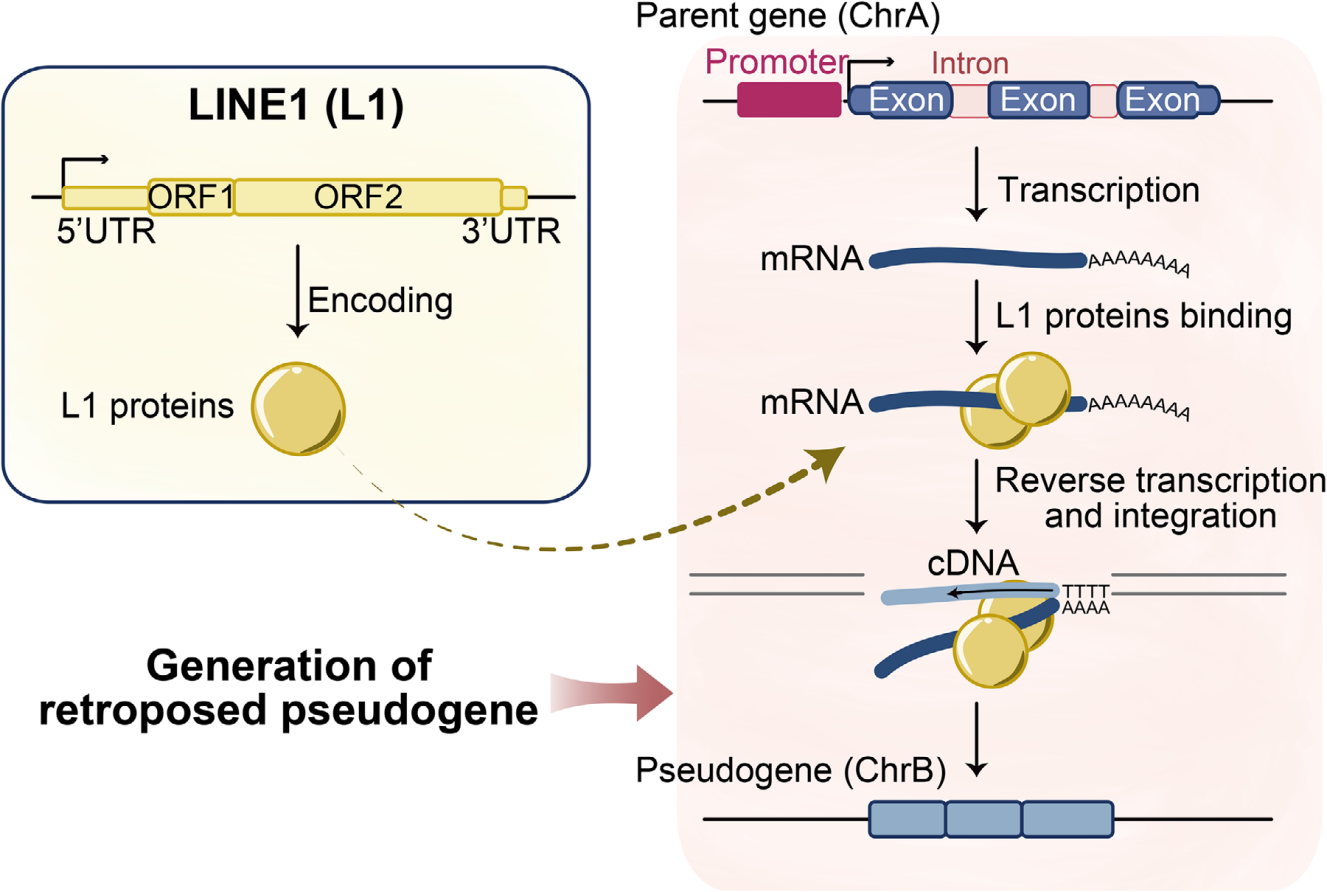
LINE1‐mediated retroposed pseudogene formation. LINE1‐encoded proteins (L1 proteins), including nucleic‐acid chaperone, reverse transcriptase, and endonuclease, bind gene transcripts, cleave genomic DNA, and integrate cDNA into the genome, resulting in intron‐less retroposed pseudogenes typically on different chromosomes from parental genes.

We systematically compare the fundamental distinctions between retroposed and duplicated pseudogenes, highlighting key differential characteristics in sequence architecture and genomic distribution patterns. Retroposed pseudogenes, generating through LINE1‐mediated retrotransposition (Figure 2), typically exhibit intron‐less structures due to mRNA intermediate processing, truncated sequences averaging ∼1.5 kb in length, frequent polyA tract remnants, and absence of parental gene regulatory elements, while duplicated pseudogenes generally maintain full‐length genomic organization including intronic regions and potential retention of upstream regulatory sequences (e.g., promoters, enhancers). Regarding genomic distribution, retroposed pseudogenes demonstrate insertional bias toward regions with open‐chromatin and actively expressed genes, random genomic dispersion mediated by retrotransposase activity, and transchromosomal localization with parental genes (e.g., *PTEN* on 10q23.31 versus *PTENP1* on 9p13.3) [36, 43, 44]. Whereas duplicated pseudogenes originate through non‐allelic homologous recombination (NAHR) or replication errors and display paralogous chromosomal clustering (e.g., *HBB*/*HBBP1* in the β‐globin locus on 11p15.4), illustrating that distinct molecular origins may fundamentally determine pseudogene architecture, genomic distribution patterns, and even regulatory potential in mammalian genomes. Moreover, retroposed pseudogenes in the human genome demonstrate pronounced primate‐specificity, particularly in the New World and Old World monkey lineage [32, 33]. In contrast, duplicated pseudogenes show relatively lower species specificity, with 40% sequence conservation between human and mouse genomes (versus 10% for retroposed pseudogenes) [3].

In conclusion, understanding the distinct characteristics of each pseudogene type, including origin, structure, and evolutionary trajectory, is essential for accurately annotating genomes and interpreting their potential biological roles. Future studies leveraging long‐read sequencing and functional genomics will further refine these classifications and reveal novel subclasses.

## 3 Functional Pseudogenes

Once considered nonfunctional genomic relics or molecular “fossils”, pseudogenes, defective paralogs of protein‐coding genes, are now gradually recognized as dynamic contributors to gene regulation. Emerging evidence demonstrates that pseudogenes can regain functional activity through diverse mechanisms [45, 46, 47, 48]. Certain pseudogenes not only transcribe regulatory non‐coding RNAs but also encode functional truncated or full‐length proteins, challenging the traditional view of their functionlessness theory. And even transcriptionally silent, pseudogenes may exert regulatory functions through their DNA sequences. These functional repertoires highlight pseudogenes as versatile genomic elements capable of influencing gene expression networks, phenotypic plasticity, and evolutionary innovation. Herein, we systematically elucidate the molecular mechanisms underlying pseudogene reactivation and their diverse functional repertoires in genomic regulation.

### 3.1 Reactivation of Pseudogenes

**FIGURE 3:**
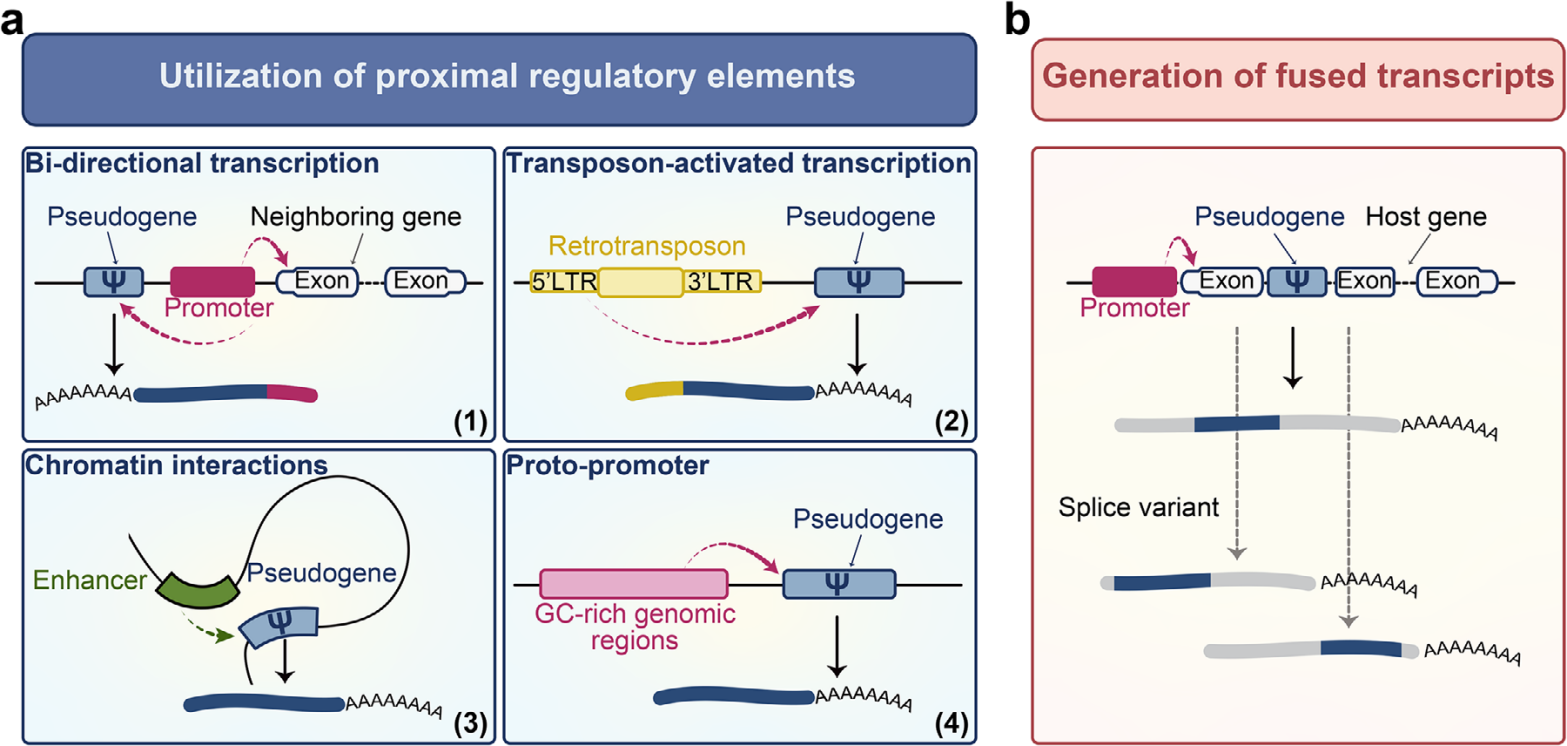
Pathways of pseudogene reactivation. (a) Transcriptional reactivation of pseudogenes is facilitated by various pre‐existing regulatory elements, including: (1) promoters from adjacent genes; (2) promoters derived from nearby transposable elements; (3) enhancers engaging in long‐range chromatin interactions; and (4) GC‐rich regions with intrinsic promoter potential. (b) pseudogenes derived fusion transcripts formed via co‐splicing with host gene exons.

Although the majority of pseudogenes in the human genome arise from retrotransposition events and therefore lack native promoter elements, their integration into the genome demonstrates significant non‐randomness, strongly influenced by the epigenetic landscape of insertion sites. These sequences exhibit a pronounced preference for open chromatin regions, enriched with regulatory signatures such as DNase I hypersensitive sites, histone modifications associated with active transcription (e.g., H3K4me3, H3K27ac), and binding sites for transcription factors [36, 43, 44]. Such positioning supports their transcriptional activation through various mechanisms: certain pseudogenes integrate within intronic or exonic regions of functional genes and hijack endogenous promoters; others exploit proximal regulatory architectures, including bidirectional promoters from adjacent genes [36, 46, 49] or promoters contributed by retrotransposons like LTRs of endogenous retroviruses (Figure 3a) [36, 46, 50]. Additionally, distal enhancers can activate pseudogene expression via long‐range chromatin interactions (Figure 3a) [36]. It is also noteworthy that the GC‐rich genomic environments often selected by retrotransposed pseudogenes may possess intrinsic promoter‐like features (Figure 3a) [51, 52]. Thus, the transcriptional competence of pseudogenes emerges from the synergistic interplay between their structural preservation and the accessibility of local and distal regulatory elements, underscoring their potential roles in gene regulatory networks.

Beyond transcriptional reactivation, pseudogenes can form fusion transcripts with adjacent or host genes via canonical or alternative splicing, repurposing their sequences into chimeric RNAs with novel functions (Figure 3b) [36, 53, 54, 55, 56]. For instance, in prostate cancer, the pseudogene *KLKP1* splices into the *KLK4* transcript, generating an oncogenic fusion RNA [53, 57]. Alternatively, pseudogenes derived exons can introduce new protein domains, as demonstrated by a *DPH3P1*‐containing *BRCA1* splice variant that produces a truncated protein with functional properties [20, 58]. These mechanisms highlight the expanding repertoire of pseudogenes driven by transcript diversification and its biological significance.

In summary, accumulating evidence demonstrates that pseudogenes are far from being genetically inert. Through either context‐dependent reactivation (such as co‐opting neighboring regulatory elements) or chimeric transcript formation with neighboring or host genes, some pseudogenes escape transcriptional silencing and may acquire novel biological functions.

### 3.2 Major Regulatory Pathways of Pseudogenes

#### 3.2.1 Noncoding Mechanisms

**FIGURE 4:**
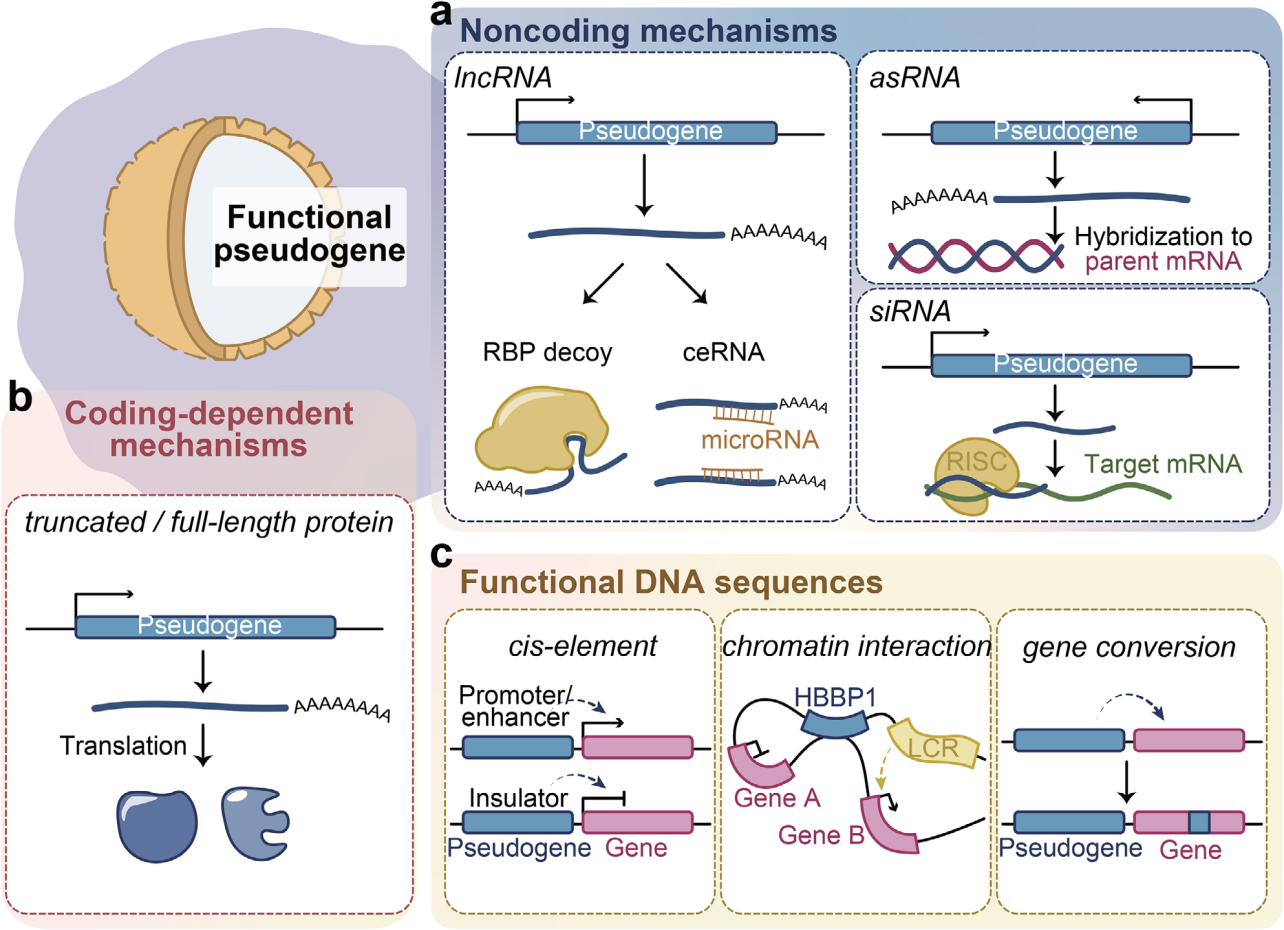
Main functional mechanisms of pseudogenes. (a) Noncoding‐dependent roles: long non‐coding RNA (lncRNA), antisense RNA (asRNA), or small interfering RNA (siRNA) production. (b) Coding‐dependent roles: truncated or full‐length protein translation. (c) DNA sequence‐mediated functions: functioning as a cis‐regulatory element (left), chromatin interactions by forming loops (middle) or gene conversion (right).

Emerging research has established that certain pseudogenes can be transcriptionally reactivated under specific cellular contexts, producing diverse noncoding RNAs that play multifaceted regulatory roles in biological processes. These pseudogenes derived transcripts exhibit remarkable functional versatility through several well‐characterized mechanisms (Figure 4a).

First, transcripts of pseudogenes may act as microRNA sponging and possess competing endogenous RNA (ceRNA) activity [59, 60, 61]. Pseudogenes derived lncRNAs frequently function as molecular decoys that sequester microRNAs, thereby modulating the expression of their parental genes, such as *PTENP1* transcripts competitively bind the *PTEN*‐targeting microRNA (miR) 19/20/21 families, elevating *PTEN* mRNA levels and suppressing tumorigenesis [14], and *BRAFP1*‐derived lncRNAs sponge microRNA, derepressing *BRAF* expression and activating MAPK signaling [11]. Moreover, pseudogenic lncRNAs can directly bind proteins to alter their activity or localization, including *HMGA1‐p* lncRNA interacts with the RNA‐stabilizing complex αCP1, destabilizing *HMGA1* mRNA [62], *RNA5SP141* binds RIG‐I during herpesvirus infection, triggering interferon production [63], and so on. Bidirectional transcription generates antisense RNAs (asRNAs) with distinct functions, and pseudogenes also can encode antisense RNAs. *PTENP1*‐AS isoforms regulate *PTEN* mRNA nuclear export and stability [64], *OCT4pg5*‐AS recruits EZH2 to the OCT4 promoter [65], silencing its expression, and *NOS*‐AS inhibits translation of neuronal nitric oxide synthase via RNA‐RNA duplex formation [66]. In addition, some pseudogenes have been found to be able to transcribe endogenous small interfering RNAs (siRNAs) and participate in the process of mRNA degradation, and pseudogenes such as *ψPPM1K*, *ATP8A2P1*, and *CNN2P1* inhibit the expression of the parent gene by transcribing siRNAs [67, 68, 69, 70].

In summary, current research has confirmed that pseudogenes derived transcripts exert regulatory functions primarily through interactions with microRNAs, proteins, or mRNAs. Due to their sequence similarity with parental genes, pseudogene transcripts preferentially target and modulate the expression of their parental genes through bidirectional regulatory mechanisms [71]. Pseudogenes can either upregulate or downregulate parental gene expression depending on their molecular mode of action. Positive regulation generally occurs when pseudogenes function as competing endogenous RNAs that sequester microRNAs targeting the parental transcript, or when they competitively bind RNA‐destabilizing proteins, thereby stabilizing parental mRNA and enhancing its expression. Conversely, negative regulation results from pseudogenes’ competition for binding to RNA‐stabilizing proteins or their generation of endogenous siRNAs that direct the cleavage and degradation of parental transcripts. This dual regulatory capacity enables pseudogenes to serve as precise molecular switches for fine‐tuning parental gene expression levels, contributing to the maintenance of cellular homeostasis and the coordination of complex biological processes. The functional outcome, activation or repression, is determined by the specific interaction partners and molecular context of each pseudogenes parental gene pair, highlighting the sophisticated regulatory potential of these once‐considered “genomic fossils”.

#### 3.2.2 Coding‐Dependent Mechanisms

While pseudogenes have been primarily characterized as sources of regulatory noncoding RNAs, accumulating evidence reveals that a biologically significant subset even retains protein‐coding potential, producing functional micropeptides and full‐length proteins that contribute to diverse physiological and pathological processes (Figure 4b). These protein‐coding pseudogenes represent a representative exception to the traditional view of pseudogenes as nonfunctional relics [72, 73, 74, 75, 76, 77].

The most typical examples demonstrate how pseudogenes derived proteins can modulate their parental gene functions through precise molecular mechanisms. The *SRGAP2C* pseudogene, for instance, encodes a truncated version of the synaptic plasticity regulator *SRGAP2* that acts as a dominant‐negative inhibitor by forming nonproductive heterodimers with the full‐length parental protein [15, 78]. This interaction may cause neoteny during spine maturation and increase the density of longer spines, potentially contributing to human‐specific brain development patterns. Similarly, the *NOTCH2NL* gene family produces proteins that enhance cortical neurogenesis by competitively binding the NOTCH pathway inhibitor DELTA‐LIKE1, thereby amplifying NOTCH signaling in neural progenitor cells [17, 79]. These cases illustrate how pseudogenes encoded proteins can evolve dosage‐sensitive regulatory relationships with their parental genes. Beyond neurodevelopmental roles, several oncogenic pseudogenes derived proteins have been identified. The *POU5F1B* pseudogene encodes a protein resembling the OCT4 transcription factor that cooperates with MYC signaling in gastric cancer, with overexpression correlating with poor prognosis [80]. Similarly, *NANOGP8* produces a protein that maintains stem‐like properties in cancer cells, potentially explaining its elevated expression in aggressive tumors [81]. Other examples include *GJA1P1*, which may encode a gap junction protein that suppresses tumor cell proliferation [82]. Besides all, the coding products of pseudogenes can be detected in other systems, such as *PGK2*, despite exhibiting canonical features of retrotransposition‐derived sequences, also maintain an uninterrupted open reading frame and demonstrate functional testis‐specific expression [12, 13]. Beyond human tissues, protein products derived from pseudogenes have also been identified in other species. For instance, the murine pseudogene *Pfh8‐ps*, which retains an intact coding sequence, gives rise to the PHF8‐PS protein [76]. This protein has lost the histone demethylase activity characteristic of its parental protein but localizes to the cytoplasm. Through interactions with prefolding and other co‐factors, it may participate in a previously unrecognized pathway involved in protein folding.

In brief, although protein‐coding pseudogenes represent a relatively small proportion of the total pseudogene repertoire, emerging evidence demonstrates their capacity to participate in critical biological processes through diverse molecular mechanisms. These functional pseudogenes encoded products often exhibit exquisite specificity in their regulatory roles, frequently targeting pathways associated with their parental genes.

#### 3.2.3 Functional DNA Sequences of Pseudogenes

Beyond their well‐characterized roles in RNA‐based regulation and potential protein‐coding activities, recent studies have revealed that pseudogenes can also function at the DNA level through mechanisms such as acting as cis‐regulatory elements, participating in chromatin‐mediated gene regulation, and facilitating non‐allelic homologous recombination (gene conversion) (Figure 4c). While less extensively studied than their transcriptional activities, these DNA‐level functions represent important and underappreciated aspects of pseudogene biology.

First, certain pseudogene sequences can function as cis‐regulatory elements. Features inherited from their parental genes, such as GC‐rich sequences and CTCF binding sites, can exhibit promoter, enhancer, or insulator activity, enabling them to participate in the regulation of neighboring gene expression [36, 47, 68, 83, 84]. Some pseudogenes may even contribute to mediating long‐range chromatin interactions, exemplified by the *HBBP1* pseudogene, which facilitates the fetal‐to‐adult hemoglobin switch by physically interacting with the locus control region (LCR) through chromatin looping, thereby modulating globin gene expression during erythroid development [85]. This singular reported case suggests that pseudogenes may serve as architectural elements in genome organization, potentially influencing gene expression through long‐range chromatin contacts, though the generalizability of this phenomenon requires further investigation. More extensive studies are the role of pseudogenes in disease pathogenesis through gene conversion events, where non‐allelic homologous recombination between pseudogenes and their functional parental genes transfers deleterious mutations [86]. This mechanism underlies numerous disorders, including hereditary pancreatitis caused by *PRSS3P2‐PRSS3* conversions, congenital adrenal hyperplasia resulting from *CYP21A2P‐CYP21A2* recombination, and various forms of inherited cataracts (*CRYBP1‐CRYBB2* conversions) [87, 88, 89]. These pathogenic gene conversion events typically occur due to the high sequence similarity between pseudogenes and their functional counterparts, with conversion tracts ranging from 20 bp to several kilobases.

The relative paucity of research on pseudogene chromatin interactions compared to gene conversion events highlights an important gap in our understanding of pseudogene biology, suggesting that future studies employing techniques like Hi‐C, ChIA‐PET, and CRISPR‐based chromatin manipulation could uncover additional examples of pseudogenes functioning as regulatory DNA elements [90, 91, 92]. Together, these DNA‐level functions expand the functional repertoire of pseudogenes beyond their well‐characterized RNA‐based activities and underscore their multifaceted roles in both normal biology and disease pathogenesis.

### 3.3 Dual Identities in Physiological and Pathological States

#### 3.3.1 Disease‐Associated Pseudogenes

As discussed, pseudogenes can exert regulatory functions at the DNA, RNA, and protein levels. Consequently, the broader biological functions and implications of these regulatory activities are receiving increasing attention. The dysregulated expression of pseudogenes in various diseases has emerged as a critical area of research, with numerous studies demonstrating their functional involvement in tumor initiation and progression through diverse molecular mechanisms [71, 93, 94, 95, 96, 97].

Among cancer‐associated pseudogenes, *PTENP1* represents a paradigm‐shifting discovery that fundamentally altered our understanding of pseudogene biology, serving as the representative well‐characterized example of a tumor‐suppressive pseudogene functioning through microRNA decoy mechanisms. The *PTEN* tumor suppressor gene has at least 17 pseudogenes in the human genome, with *PTENP1* being an extensively studied retroposed pseudogene sharing high 3’UTR sequence homology with its parental gene. *PTENP1* exerts its tumor‐suppressive function primarily by competitively binding a panel of oncogenic microRNAs, including miR‐17, miR‐19, miR‐21, miR‐26, miR‐214, and so on, that normally target *PTEN* mRNA for degradation, thereby acting as a molecular sponge that protects *PTEN* transcripts from microRNA‐mediated suppression [14]. Beyond this canonical ceRNA mechanism, *PTENP1* displays remarkable regulatory complexity through the production of two distinct antisense RNA isoforms (α and β) that differentially modulate *PTEN* expression: the α isoform mediates trans‐regulation by recruiting DNA methyltransferases to the *PTEN* promoter to epigenetically modulate its transcription, while the β isoform forms RNA‐RNA duplexes with *PTEN* mRNA to regulate its stability, nuclear‐cytoplasmic transport, and microRNA accessibility [64]. The clinical relevance of *PTENP1* is underscored by its frequent downregulation in gastric cancer, squamous cell carcinoma, and other malignancies, where reduced *PTENP1* expression correlates with decreased *PTEN* levels and worse clinical outcomes [98, 99, 100, 101, 102]. Parallel to *PTENP1*, other pseudogenes like *KRASP1* and *BRAFP1* have been implicated in cancer pathogenesis through similar ceRNA mechanisms, with *KRASP1* sponging miR‐143 and let‐7 family to protect *KRAS* mRNA [14] and *BRAFP1* enhancing oncogenic signaling by sequestering microRNAs that target *BRAF* [11, 103]. The pervasive dysregulation of pseudogenes across cancer types—with some functioning as tumor suppressors (e.g., *PTENP1*) and others as oncogenic drivers (e.g., *KRASP1*, *BRAFP1*)—highlights their importance as a novel class of cancer genes. These findings have catalyzed a new era of pseudogene research in oncology, suggesting their potential as diagnostic biomarkers, therapeutic targets, and mediators of treatment resistance. Future studies integrating single‐cell analyses, CRISPR screening, and clinical correlative studies will be essential to fully exploit the translational potential of cancer‐associated pseudogenes.

Beyond transcriptional dysregulation, pseudogene copy number variations (CNVs) have emerged as significant contributors to human disease pathogenesis, with the *NOTCH2NL* locus serving as a paradigmatic example of how pseudogene structural variations can exert bidirectional effects on neurodevelopment [79, 104]. The *NOTCH2NL* gene family, as we mentioned above, derived from partial duplications of the *NOTCH2* receptor gene, exhibits dosage‐sensitive effects on cortical development, where increased copy numbers are associated with autism spectrum disorder and macrocephaly, while deletions correlate with microcephaly and schizophrenia. This dual pathology stems from *NOTCH2NL*’s role in modulating NOTCH signaling dynamics‐copy number gains amplify cortical progenitor expansion through enhanced *NOTCH* activation, whereas losses impair neurogenesis due to insufficient NOTCH pathway stimulation [79]. More broadly, the *NOTCH2NL* paradigm underscores the importance of systematically evaluating pseudogene CNVs across the genome, particularly for human‐specific duplicates that may underlie both our unique cortical expansion and associated neuropsychiatric vulnerabilities.

The association between pseudogenes and disease is not limited to humans but also extends to susceptibility traits in other species. A notable example is found in canids, where numerous retrocopies (a class of pseudogenes) have been identified [26]. Among these, a retrocopy of fibroblast growth factor 4 (*FGF4*) shows a strong association with a chondrodysplasia phenotype (characterized by short limbs) and is a major genetic factor underlying both this morphology and a high prevalence of intervertebral disc disease (IVDD) in certain dog breeds [105, 106]. This case illustrates how pseudogenes can contribute to disease susceptibility across species.

#### 3.3.2 Physiological Roles of Pseudogenes

In addition to being associated with disease progression, current research has also established that pseudogenes play critical regulatory roles during normal development, with several well‐characterized examples demonstrating their involvement in stem cell maintenance, development, cellular immunity, and so on.

Our previous research has uncovered a pseudogenes expression network during human early embryonic development, where we systematically identify 140 functionally active pseudogenes that exhibit stage‐specific expression patterns across critical developmental windows, and these embryonic‐expressed pseudogenes display critical regulation functions in both naive (pre‐implantation) and primed (post‐implantation) pluripotency states [107]. Meanwhile, in addition to the above‐mentioned DNA sequences of *HBBP1* involved in the formation of chromatin interactions, we also found that the transcription product of *HBBP1* can participate in the process of erythropoiesis by competitively binding to hnRNPA1 with *TAL1* mRNA, suggesting the critical role of *HBBP1* in the normal development of the human blood system [16]. Pseudogenes may also participate in immune responses, as evidenced by the identification of 54 pseudogenes derived from lncRNAs within the TNFα‐induced inflammatory signaling pathway in mice, which demonstrate precisely regulated expression patterns [108].

In summary, although research on pseudogenes in normal physiological processes remains relatively limited compared to their pathological roles, their crucial regulatory functions cannot be overlooked. The dual functionality of pseudogenes, operating in both pathological and physiological contexts, renders certain pseudogenes indispensable within the human genome. Moreover, in recent years, high‐throughput sequencing technologies have revealed the phenomenon of “pervasive transcription” in mammalian genomes, yielding vast quantities of non‐coding RNAs with unknown functions, often referred to as “dark matter RNA” [109, 110]. This discovery has sparked considerable debate within the scientific community regarding whether these widespread transcriptional outputs represent regulatory molecules with specific biological roles or merely reflect non‐functional “transcriptional noise”. The transcripts derived from pseudogenes, which are the focus of this review, lie at the heart of this controversy. Although pseudogenes originate from inactivated gene copies and are often regarded as evolutionary by‐products, accumulating evidence demonstrates that some pseudogene transcripts play crucial regulatory roles through mechanisms such as ceRNA activity. Therefore, elucidating the functions of pseudogenes not only advances our understanding of pseudogenes themselves but also provides important insights into the biological significance of pervasive transcription.

## 4 Roles of Pseudogenes in Evolution

As duplicated sequences are highly similar in sequence to their parental genes, pseudogenes can be functionally reactivated under specific conditions to exert regulatory roles at multiple molecular levels. While parental genes represent the predominant targets of pseudogenes mediated regulation, emerging evidence reveals their involvement in broader signaling networks as well. Due to their diverse mechanisms of action and wide range of molecular targets, pseudogenes demonstrate the capacity to perform critical biological functions in both physiological and pathological contexts. Gene family expansion through sequence duplication represents a well‐established mechanism for functional innovation [111]. Similarly, as products of sequence duplication, the high degree of species specificity observed in human pseudogene sequences suggests that human‐ or primate‐specific pseudogenes in particular may play pivotal roles in species‐specific regulatory processes. Growing research supports this evolutionary perspective, highlighting the regulatory potential of pseudogenes in shaping species‐specific adaptations. Current findings position pseudogenes as important genomic elements that contribute to innovation through their unique regulatory capabilities.

### 4.1 Functional Pseudogenes During Evolution

#### 4.1.1 Functionalization Caused by Activated Pseudogenes

**FIGURE 5:**
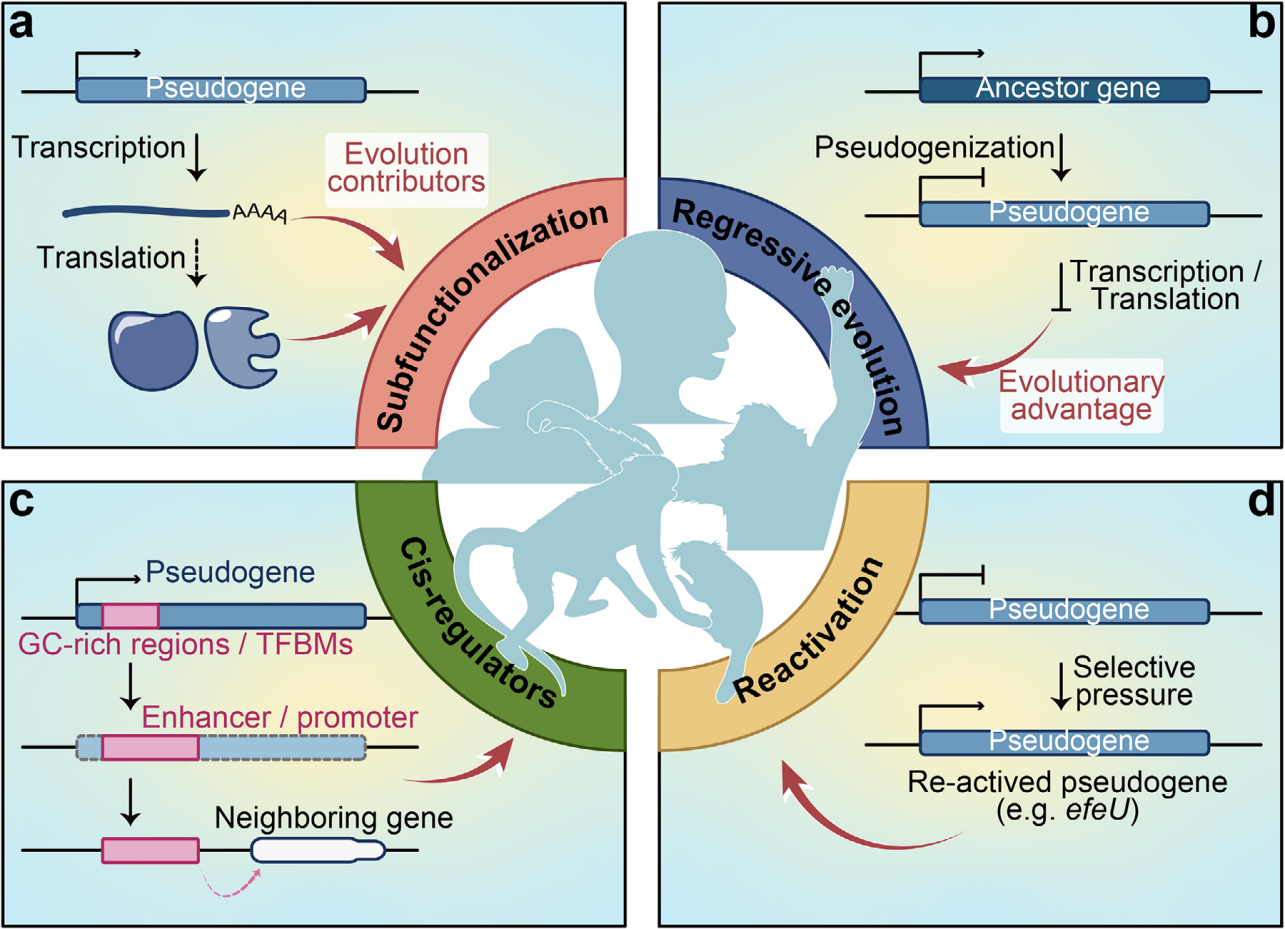
Pseudogene contributions to species evolution. (a) Novel functions from transcribed or translated pseudogenes. (b) Adaptive advantages via functional gene pseudogenization. (c) Evolution into cis‐regulatory elements such as enhancers or promoters, via GC‐rich sequences or transcription factor binding motifs (TFBMs) on pseudogenes. (d) Reactivation of pseudogenes under certain selective pressures as a genomic reservoir.

While pseudogenes are commonly thought to be retained in the genome due to their selective neutrality, growing evidence reveals that these “genomic fossils” can also acquire critical biological functions during species evolution. This occurs primarily through two evolutionary paths: the resurrection of the original function via compensatory mutations or the neofunctionalization through gain‐of‐function mutations (Figure 5a).

The pseudogenes we will discuss, *NOTCH2NL*, *HBBP1*, and *IRF5P1*, exemplify species‐specific regulatory innovations, each revealing distinct paths to functional evolution. The human‐specific *NOTCH2NL* duplicates, originating from partial duplication of the *NOTCH2* gene, exhibit dosage‐sensitive regulation of cortical neurogenesis by modulating *NOTCH* signaling dynamics, a mechanism fundamentally linked to human brain expansion [79, 17]. Notably, *NOTCH2NL* copy number variations demonstrate pleiotropic effects on human neurodevelopmental disorders. Similarly, the hemoglobin cluster pseudogene *HBBP1* has evolved human‐specific functionality in erythropoiesis through a unique RNA‐protein interaction network wherein its transcript stabilizes *TAL1* mRNA by competitively binding hnRNPA1, thereby creating a human‐specific regulatory circuit. Intriguingly, in some species, the *HBBP1* locus even retains protein‐coding capacity, suggesting that its regulatory function was remodeled during human‐specific adaptation [16]. Furthermore, the higher primate‐specific pseudogene *IRF5P1* is a chimeric element composed of sequences derived from multiple origins, likely originating approximately 60 million years ago through an ancient retroviral integration event [42]. Located within the *TRIQK* gene, it is transcribed into antisense RNA, which may bind to *IRF5* mRNA and post‐transcriptionally regulate its expression. This mechanism suggests a significant evolutionary role for pseudogenes in shaping immune system adaptations in primates.

These examples illustrate the diverse mechanistic paths by which pseudogenes acquire new functions. As introduced in Section 3, under certain conditions, pseudogenes can be reactivated to produce functional RNAs or even encode proteins. The transcriptional or translational products of pseudogenes may either exert functions analogous to those of their parental genes, such as *NOTCH2NL* and *IRF5P1*, or acquire novel biological roles, as exemplified by *HBBP1*. Regardless of the mechanism of their functional “resurrection”, these revitalized pseudogenes may contribute to adaptive evolutionary processes, thereby becoming selectively retained in the genome. In sum, pseudogenes can acquire lineage‐restricted regulatory roles through structural innovation, develop entirely new interaction networks, and become genetically embedded in species‐defining biological processes. The biomedical significance is also profound, as these species‐specific pseudogene functions simultaneously represent both evolutionary adaptations and disease association. Future research should explore whether there are more pseudogenes contributing to human‐specific or primate‐specific traits, whether such species‐specific pseudogene networks follow predictable evolutionary patterns or emerge stochastically, and how their manipulation might address human‐specific disorders.

#### 4.1.2 Evolutionary Advantages Resulted From Pseudogenization

Pseudogenes can still provide an evolutionary advantage to an organism even if it has lost its original coding function (Figure 5b). A prime example is *Xist*, essential for X‐chromosome dosage compensation in placental mammals, which evolved through the pseudogenization of the ancestral *Lnx3* protein‐coding gene [112]. Intriguingly, its transition to a noncoding regulator appears to have preceded the acquisition of X‐inactivation functions, exemplifying how pseudogenes can serve as genomic substrates for the emergence of new regulatory elements. Furthermore, the *MYH16* pseudogenization event represents a pivotal transition in human evolution, as reported by Stedman et al. [113]. The inactivation of this masticatory muscle myosin gene triggered a cascade of morphological changes, such as the decreased mechanical constraints on cranial growth resulting from reduced temporal muscle mass, thus enabling brain expansion and facilitating cognitive advancements characteristic of the Homo genus. Moreover, the pseudogenization of *ZP3R* in primates, a key gene mediating sperm‐egg adhesion, is likely linked to a relaxation of sexual selection pressure [114, 115]. During the evolution of humans and higher primates, mutations in the *GULOP* gene led to the loss of the ability to synthesize vitamin C endogenously [116, 117, 118]. Similarly, the pseudogenization of the urate oxidase (*UOX*) gene likely contributed to altered purine metabolism [119, 120]. These functional losses may represent metabolic adaptations that confer evolutionary advantages by conserving energy and optimizing resource allocation. Furthermore, in felids, the pseudogenization of the sweet taste receptor gene *Tas1r2* is likely an adaptive trait associated with their obligate carnivorous diet [121, 122].

Beyond mammals, classic examples of “loss‐of‐function” mutations and pseudogenization driving adaptive evolution are also prevalent in other metazoans. For instance, the ZRS (ZPA regulatory sequence), a critical enhancer for limb development in tetrapods, has undergone severe degeneration or even complete loss in the snake genome [123, 124]. This loss led to the termination of limb development programs in snakes, which may have facilitated adaptation for burrowing or slithering locomotion. Similarly, in fish species inhabiting dark cave environments, such as Astyanax mexicanus, visual degeneration is commonly observed [125, 126]. This trait is strongly associated with inactivating mutations in multiple eye development genes. The loss of eyesight provides significant adaptive evolutionary advantages for survival in nutrient‐poor, lightless environments.

Throughout evolution, certain genes have lost their protein‐coding capacity, while others have become transcriptionally silent. Paradoxically, such losses may themselves represent evolutionary gains. It is possible that sporadic gene inactivation events conferred adaptive advantages, such as increased brain size or more efficient metabolic pathways, thereby promoting their retention in the genome. These “regressive evolution” pathways, where loss‐of‐function mutations drive adaptive complexity, reveal pseudogenes as dual‐natured genomic elements that, while molecularly “broken” relative to ancestors, pseudogenes may still gain an evolutionary advantage for innovation. The above paradigms underscore how pseudogenization contributes to species‐defining adaptations, and it's possible that having more pseudogenes that lose their function is instead an evolutionarily better choice.

### 4.2 Evolutionary Potential of Pseudogenes as Cis‐Regulators

Regardless of whether pseudogenes can resurgence, their reintegration itself represents an expansion of the genomic repertoire [127, 128, 129]. Pseudogene integration increases sequence complexity within the genome, in which certain pseudogenes may serve as reservoirs of potential cis‐regulatory elements (Figure 5c) [36, 47, 68, 83, 84]. For instance, retroposed pseudogenes derived from protein‐coding genes may carry GC‐rich regions and transcription factor binding motifs (TFBMs), providing raw material for the evolution of promoters or enhancers. The presence of TFBMs in retroposed pseudogenes enhances their potential to evolve into enhancer‐like regions capable of modulating neighboring gene expression, and these motifs may degenerate over evolutionary time in the absence of selective pressure. Moreover, some pseudogenes can inherit evolutionarily competent promoter activity from their parental genes, as exemplified by the interaction between *PPP1R26P1* and the *RB1* locus [130]. The retrotransposition event generating *PPP1R26P1* occurred prior to the divergence of New and Old World monkeys, resulting in its integration within intron 2 of *RB1*. The *PPP1R26P1* sequence derived from exon 4 of its parental gene contains a differentially methylated CpG island that evolved promoter activity, which can serve as an alternative promoter contributing to generating novel splice isoforms for the host gene. In addition to evolving promoter activity to facilitate gene expression, pseudogenes may also function as cis‐regulatory elements in gene silencing processes. For instance, small RNAs derived from pseudogenes can recruit SETDB1 (a histone H3K9 methyltransferase), leading to trimethylation of histone H3 at lysine 9 (H3K9me3), which induces localized chromatin repression and transcriptional silencing [68].

Functioning as cis‐regulatory elements may be a key reason why some pseudogenes, despite losing protein‐coding capacity, have not been eliminated during evolution. Studies have shown that for genes with lost coding potential (GLCPs), although their ability to produce proteins is abolished, enhancer elements embedded within their genomic regions can retain critical biological functions [131]. Natural selection acts upon these enhancer sequences to maintain their regulatory roles, thereby preserving the entire locus. We propose that this evolutionary mechanism also applies to pseudogenes. As a class of GLCPs, pseudogenes may retain or even evolve regulatory elements such as promoters, enhancers, or insulators. The regulatory signals in which they participate may confer adaptive advantages during evolution, facilitating their retention in the genome.

### 4.3 Reactivation of Pseudogenes Under Selective Pressure

The above findings demonstrate that pseudogenes can serve distinct yet equally important evolutionary functions by directly participating in species adaptation as key evolutionary drivers. Some pseudogenes, even though they may not be currently indispensable for the survival of organisms, may provide an evolutionary basis for species innovation (Figure 5c). The phenomena we currently observe represent products of billions of years of evolution, yet we must recognize that evolution remains an ongoing process, so the current lack of detectable function does not preclude a sequence's potential to acquire regulatory roles under selective pressures.

A notable example in primate evolution involves the loss and functional recovery of the *IRGM* gene, a key member of the Immunity‐Related GTPase (IRG) family that defends against intracellular pathogens like Mycobacterium tuberculosis and Toxoplasma [132, 133]. After anthropoids diverged from prosimians around 50 million years ago, most IRG clusters were deleted, though *IRGM* was retained. It was later inactivated when an Alu retrotransposon disrupted its open reading frame, turning it into a pseudogene. However, a critical mutation in the common ancestor of humans and great apes restored the coding sequence by converting a stop codon into an amino acid codon, allowing functional protein production to resume. This cycle of loss and revival illustrates how evolutionary pressures, such as host‐pathogen conflicts, can drive functional innovation even after gene inactivation, highlighting the dynamic nature of genomic evolution in response to changing selective demands.

However, the regulatory systems governing gene expression in eukaryotes have evolved to be highly complex, making it experimentally challenging to verify whether certain pseudogenes could be reactivated under specific selective pressures to resume transcription or even protein production. In contrast, the comparatively simpler gene regulatory architecture of prokaryotes may provide a critical model system for investigating the molecular mechanisms and evolutionary potential of pseudogene resurrection. A compelling example emerges from bacterial systems, that under iron‐limiting conditions, *Escherichia coli* (*E. coli*) can reactivate the pseudogene *efeU* through adaptive mutations that restore its promoter activity and repair frameshift errors, reconstituting an intact open reading frame [134]. Then the resuscitated *efeU* protein forms a positive feedback loop with the entCEBA operon to enhance iron uptake efficiency. This case fundamentally demonstrates how specific environmental pressures can drive functional pseudogene resurrection through targeted genomic editing. Although the E. coli genome is less complex than the human genome, it is worth exploring whether pseudogenes in the human genome can also serve as an evolutionary “pre‐adaptive” reservoir.

In summary, we propose several principal mechanisms through which pseudogenes may contribute to species evolution. A well‐documented pathway involves the reactivation of pseudogenes, which can restore, either partially or fully, the function of their parental genes or even acquire independent regulatory roles. The transcriptional or translational products of such reactivated pseudogenes may participate in gene regulatory networks, thereby conferring evolutionary advantages. Conversely, the pseudogenization of functional genes leads to the loss of their protein‐coding or transcriptional capacity. This loss may itself represent an adaptive outcome, wherein the elimination of non‐essential genetic elements optimizes energy allocation and refines survival strategies, ultimately promoting the retention of such pseudogenes in the genome. Furthermore, we highlight additional evolutionary potentials of pseudogenes, including their roles as cis‐regulatory elements modulating the expression of other genes, as well as their capacity for functional resurrection under specific selective pressures. These observations underscore the dynamic and plastic nature of genomic evolution, illustrating how pseudogenes serve as versatile substrates for evolutionary innovation.

## 5 Technological Advances Driving the Mining of Functional Pseudogenes

As previously discussed, pseudogenes have been found to exert regulatory functions through diverse mechanisms, and their relationship with evolutionary processes is increasingly being explored. Technological advances have been indispensable in uncovering pseudogene functionality, such as the development of more accurate algorithms and the application of long‐read sequencing in pseudogene annotation. Furthermore, the rapid advancement and biological application of deep learning, particularly in genomics, transcriptomics, and evolutionary studies, are poised to revolutionize pseudogene research by improving detection, functional classification, and evolutionary annotation across species [135, 136, 137, 138, 139]. Here, we focus on and discuss several transformative technological developments that have recently contributed most significantly to the field, including the use of long‐read sequencing for accurate quantification of pseudogenes, the integration of single‐cell omics in pseudogene studies, the potential of large‐scale comparative genomics, and the facilitation of functional validation through gene editing technologies.

### 5.1 Application of Long‐Read Sequencing Technologies in Pseudogene Quantification

Accurately quantificating pseudogenes derived transcripts is crucial for elucidating their biological functions. Although parallel RNA sequencing (RNA‐seq) can identify lncRNA transcripts, the short‐read fragments obtained may be insufficient to distinguish sequence differences between pseudogenes and their parental genes [140, 141, 142]. Specialized pipelines have been developed to focus only on reads from divergent regions of pseudogenes [143], but for evolutionarily younger pseudogenes, RNA‐seq‐captured sequences may not yet exhibit sufficient divergence from their parental genes. A notable example involves *GBA1*, a gene whose mutations cause Gaucher disease and represent the most significant genetic risk factor for Parkinson's disease. Its highly homologous pseudogene, *GBAP1*, poses major mapping challenges: over 50% of short RNA‐seq reads cannot be uniquely aligned to either *GBA1* or *GBAP1*, confounding accurate expression quantification and significantly complicating transcriptomic studies at this locus [144]. The emergence of long‐read sequencing technologies fundamentally addresses this limitation [145, 146, 147, 148, 149]. By spanning multiple divergent sites within a single read, long‐read platforms enable unambiguous assignment and precise quantification of transcripts originating from *GBA1* versus *GBAP1*.

At a broader scale, PacBio long‐read cDNA sequencing applied to normal human tissues and cancer cell lines has led to the discovery of hundreds of novel transcribed pseudogenes, some of which even retain protein‐coding potential [50, 150, 151]. Integrating long‐read sequencing technologies to identify dynamically expressed pseudogenes throughout mammalian development, and to further investigate their functional and evolutionary characteristics, represents a major application of these advances in pseudogene research. Thus, despite possessing the most complete eukaryotic transcriptome annotation available, human reference genomes continue to reveal numerous previously unannotated genes through long‐read sequencing, underscoring both the incompleteness of current annotations and the transformative potential of emerging technologies.

In sum, long‐read sequencing technologies (e.g., PacBio HiFi and Oxford Nanopore) overcome key limitations of short‐read approaches in pseudogene research by providing complete, unambiguous sequence contexts. They enable accurate detection of pseudogene‐specific features and resolve complex genomic regions, including segmental duplications and structural variations. These advances support precise classification of pseudogenes and reveal evolutionary dynamics in repetitive gene families. Additionally, native epigenetic profiling (e.g., 5mC detection via Nanopore) links structural variation to regulatory mechanisms, offering multidimensional insights into pseudogene silencing and functional evolution. Long‐read sequencing thus represents a transformative tool for decoding pseudogene biology and genome evolution. Certainly, it is undeniable that long‐read sequencing currently suffers from limited sequencing depth, which may hinder the detection of pseudogenes with low expression abundance. Therefore, when performing quantitative analysis of pseudogenes in research, a combined approach utilizing both long‐read and short‐read sequencing can be adopted. This strategy involves applying pseudogene‐specific discrimination methods during short‐read data analysis, while simultaneously leveraging long‐read sequencing to reconfirm pseudogene fragments that exhibit high sequence similarity to their parental genes.

### 5.2 Single‐Cell Technologies Revealing Pseudogene Expression and Function

Long‐read sequencing enhances the accuracy of gene detection and quantification, while single‐cell sequencing technologies enable the resolution of cell type‐specific expression patterns across diverse tissues, developmental stages, and disease conditions at unprecedented resolution, effectively overcoming the limitations of cellular heterogeneity inherent in conventional bulk sequencing approaches. These advances have also significantly propelled research in the field of pseudogenes.

For instance, through the application of single‐cell sequencing, the pseudogene *mOct4pg9* was identified as being specifically highly expressed in mouse neural stem/progenitor cells [152]. Its transcribed non‐coding RNA was shown to play a critical role in impairing neural stem cell (NSC) function and disrupting brain development following fetal ethanol exposure. Another example involves *CLEC9A*, a gene encoding a key receptor protein essential for cross‐presentation in mammalian immunity. In Sus scrofa, this gene has been pseudogenized due to mutation. Single‐cell sequencing revealed specific expression of this pseudogene primarily in dendritic cells, suggesting a potential association with dendritic cell maturation and functional status [153]. Furthermore, the expanding repertoire of surface markers in single‐cell studies now includes certain pseudogenes, indicating that not only can their expression be detected via single‐cell technologies, but some pseudogenes derived transcripts may also serve as markers for specific cell types [154].

In summary, single‐cell sequencing technologies enable the identification of pseudogenes with cell‐specific expression patterns and facilitate the analysis of their cellular and spatial expression heterogeneity. These capabilities provide critical insights into the functional roles and regulatory mechanisms of pseudogenes, thereby advancing our understanding of their contributions to development, homeostasis, and disease.

### 5.3 Charting the Pseudogene Landscape via Large‐Scale Comparative Genomics Studies

Fueled by advances in the depth and breadth of sequencing technologies, large‐scale comparative genomics studies are flourishing. These efforts, leveraged by consortium projects like ENCODE, GTEx, TCGA, and gnomAD, provide public data resources that enable the systematic, system‐level investigation of pseudogene biology.

For example, epigenomic analyses from ENCODE have revealed that certain pseudogene loci exhibit activating histone marks, such as H3K4me3 and H3K27ac, indicative of active enhancer or promoter activity [109]. Their chromatin accessibility profiles and transcription factor binding patterns further suggest that some pseudogenes may function as independent regulatory elements. Leveraging multi‐tissue RNA‐seq data from the GTEx project, it is now feasible to construct a comprehensive expression atlas of pseudogenes, identify those with tissue‐specific expression, and infer biological functions through co‐expression network analyses that reveal coordinated activities between pseudogenes and protein‐coding genes [155]. Cancer genomics initiatives like TCGA have further propelled pseudogene research into the clinical arena [156]. Pan‐cancer analyses comparing pseudogene expression in tumors versus normal tissues have identified cancer‐specific pseudogenes, many of which show correlations with cancer subtypes, tumor stage, and patient prognosis. Population genomics resources such as gnomAD enable the assessment of evolutionary constraints on pseudogene regions [157]. Although pseudogenes generally experience weaker purifying selection than coding genes, some exhibit significant mutational constraint across human populations, suggesting potential functional importance.

In summary, large‐scale comparative studies leveraging these rich datasets are making substantial contributions to uncovering the functional landscape of pseudogenes and elucidating the principles governing their biological and clinical relevance.

### 5.4 Functional Validation of Pseudogenes via Gene Editing

Advances in omics technologies are providing an unprecedented, comprehensive view of pseudogenes, and the application of gene editing tools is enabling their precise manipulation.

When performing genetic manipulations to investigate pseudogene function, due to sequence similarity between pseudogenes and their parental genes, precisely manipulating pseudogene expression without off‐target effects on parental genes remains challenging. Traditional RNA interference (e.g., small interfering RNA) employs short fragments that may not reliably target pseudogenes specific divergent regions, leading to high off‐target risks. The emergence of CRISPR technology provides a solution for DNA‐level pseudogene targeting, enabling knockout via pseudogenes specific flanking sequences or expression modulation (e.g., CRISPR interference, CRISPR activation) by targeting promoter regions [158, 159, 160, 161]. Employing CRISPR‐based knockout or knockdown strategies, such as targeting *NANOGP8* for deletion or *PTENP1* for transcriptional repression, enables functional investigation of pseudogenes while minimizing off‐target effects on their parental genes due to improved guide RNA specificity [162, 163]. Furthermore, large‐scale CRISPR screens have been increasingly applied to systematically identify functional pseudogenes across the genome. A recent study has successfully used CRISPR libraries to knock down a subset of pseudogenes, demonstrating the feasibility of CRISPR‐based pseudogene interference [164].

When applying CRISPR technology, it is essential to design gRNAs specifically targeting sequence divergence sites between the pseudogene and its parental gene, so as to minimize off‐target effects and unintended disruption of the functional parental gene. Suitable target sites may include exon‐intron junctions, which are absent in the parental gene due to the intron‐less nature of retroposed pseudogenes or external sequences such as upstream regulatory regions unique to the pseudogene locus. However, for pseudogenes integrated into host genes or sharing cis‐regulatory elements with nearby genes, DNA‐level manipulations may disrupt the integrity or expression of neighboring genes. Thus, when performing CRISPR, it is essential to exclude interference with adjacent gene functions. For CRISPR library screens targeting functional pseudogenes, optimal candidates may be either host‐free, integrate into intronic regions, or sufficiently distant from neighboring genes to minimize unintended effects. Another important experimental consideration is that when using Cas9 to target the parental gene, the high sequence similarity between pseudogenes and their parental counterparts may lead to gene conversion events during DNA repair, where the broken parental allele is repaired using the pseudogene sequence as a template [165]. Conversely, a similar mechanism may occur during pseudogene knockout, where the parental gene sequence is used for repair, potentially restoring functionality. To enhance the efficacy of pseudogene knockout, one may introduce an exogenously designed homology‐directed repair (HDR) template to introduce out‐of‐frame insertions or deletions. This strategy can induce premature termination codons and trigger nonsense‐mediated mRNA decay (NMD), ultimately achieving stable knockout.

In summary, although several technical challenges warrant careful attention in CRISPR‐based editing of pseudogenes, the technology remains highly advantageous due to its precision and ability to minimize confounding effects from homologous parental genes.

## 6 Conclusions and Perspective

An increasing number of studies have revealed the regulatory functions that pseudogenes may exert at various levels. As products of species evolution, the functional roles of pseudogenes are also likely to participate in evolutionary regulation. The growing understanding of pseudogene functions has led us to question whether the traditional binary classification of functional genes is still suitable for distinguishing so‐called pseudogenes from true genes. For example, the functional pseudogene *NOTCH2NL*, as mentioned above, despite its typical sequence truncation and pseudogenic origin, has been redefined as a protein‐coding gene due to its known function. Where, then, lies the boundary between pseudogenes and true genes? Even if they currently lack function, pseudogenes may be reactivated under special conditions—so does that mean they can then be called protein‐coding genes? We argue that the functional manifestation of pseudogenes is context‐dependent, making them more representative of a potential state rather than a simple gene‐type classification. Because labeling as pseudogenes, they are often excluded from genomic analyses and functional screenings, leading to the potential oversight of many functional individuals. As proposed by Seth W. Cheetham et al. [18], retroposed pseudogenes, which are generated through retrotransposition, can be collectively termed “retrocopy”, while duplicated pseudogenes, arising from DNA segment duplication, may be referred to as “gene copy” or “paralogue”. Current gene‐type classifications may no longer serve as criteria for assessing functional potential; instead, auxiliary genotyping based on the mechanism of origin may provide more insight into their sequence composition, relative conservation, and even functional modes [5].

The functional roles of pseudogenes and their potential resurrection under selective pressures have reshaped our understanding of the mechanisms driving their persistence and functionalization. The evolutionary trajectories of pseudogenes are influenced by a dynamic interplay between neutral evolution and positive selection, which may operate on different timescales. Initially, pseudogenes accumulate mutations neutrally in the absence of functional constraints. However, under shifting evolutionary pressures, such as pathogen invasion or metabolic changes, a once‐neutral pseudogene may be co‐opted for a novel function, leading to the onset of positive selection that refines its sequence for this new role. This transition from a neutral “genomic fossil” to a functional element underscores the adaptive potential of pseudogenes. Beyond sequence‐level evolution, structural variation (SV), including segmental duplications and copy number variations (CNVs), significantly accelerates pseudogene birth and death rates, which exerts a profound and dramatic impact on the abundance of pseudogenes [166]. SV generates redundant gene copies that serve as substrates for evolutionary innovation, while deletions within CNVs can eliminate pseudogenes containing segments, increasing their genomic turnover. Divergence in SV patterns among species partly explains the observed variation in pseudogene repertoires across taxa. The computational simulation framework established in previous work also provides a highly flexible and powerful tool for investigating the origin and evolution of gene families, reflecting the processes of gene “birth” and “death”, thereby enabling more nuanced studies of pseudogene sequence evolution [167]. Taken together, pseudogenes serve as crucial sources of genomic plasticity. They provide a reservoir of low‐constraint sequences that facilitate rapid functional exploration, complementing the more rigid optimization of protein‐coding genes and offering dynamic modules for evolutionary innovation.

As previously introduced, existing studies have revealed that individual pseudogenes may perform important regulatory functions at various molecular levels and under different physiological states. Species‐specific pseudogene functions may even contribute to evolutionary processes. Therefore, investigating whether more pseudogenes participate in biological activities and potentially contribute to species evolution, in other words, systematically studying and elucidating the functional roles of the pseudogene population, is of critical importance. For example, profiling the expression patterns of pseudogenes across different developmental stages and physiological states to identify differentially expressed pseudogenes; or comparing orthologous developmental processes (e.g., neurodevelopment, embryogenesis) across species to explore whether pseudogenes may be associated with species‐specific phenotypic differences. Beyond functional exploration, the molecular mechanisms underlying pseudogene regulation remain largely unclear. While some functions at the DNA, RNA, and protein levels are known, the vast number of pseudogenes still lack well‐defined mechanisms. Could they function at other regulatory levels? These are compelling questions for further investigation. Meanwhile, it should be noted that this review primarily focuses on pseudogenes derived from protein‐coding genes, whereas the study of pseudogenes originating from non‐coding RNA genes (e.g., miRNA‐derived pseudogenes, lncRNA‐derived pseudogenes) presents more distinct and considerable challenges. Unlike protein‐coding pseudogenes, which can often be readily identified as loss‐of‐function through frameshift mutations or premature stop codons, confirming the loss of function in a non‐coding RNA pseudogene typically requires extensive experimental validation. This often includes functional characterization of homologous loci in other species to demonstrate the loss of specific regulatory activities, among other approaches. The current lack of such data in the literature has resulted in the underrepresentation of non‐coding pseudogenes in evolutionary studies. The identification of pseudogenes derived from different types of parent genes remains a significant challenge in the field.

Thus, pseudogenes are, in fact, crucial sequence resources for species evolution. Although current research still faces challenges, we believe that, with advancing technologies and deepening understanding, the important roles of pseudogenes will be further unveiled.

## Conflicts of Interest

The authors declare no conflicts of interest.
